# Polypyrrole-based nanocomposites doped with both salicylate/molybdate and graphene oxide for enhanced corrosion resistance on low-carbon steel

**DOI:** 10.1080/15685551.2023.2220529

**Published:** 2023-06-10

**Authors:** Ha Manh Hung, Tran Minh Thi, Le Van Khoe, Le Minh Duc, Hoang Thi Tuyet Lan, Lai Thi Hoan, Vu Thi Xuan, Nguyen Thi Bich Viet, Ngo Xuan Luong, Nguyen Thuy Chinh, Thai Hoang, Vu Thi Hương, Vu Quoc Trung

**Affiliations:** a Faculty of General Education, Hanoi University of Mining and Geology, Hanoi, Vietnam; b Institute for Theoretical and Applied Research, Duy Tan University, Hanoi, Vietnam; c Faculty of Nature Science, Duy Tan University, Da Nang, Vietnam; d Faculty of Natural Sciences, Hong Duc University, Thanh Hoa, Vietnam; e Branch of National Institute of Occupational Safety and Health & Environmental Protection in Central of Vietnam, Hai Chau, Da Nang, Vietnam; f Faculty of Basic Sciences, University of Transport and Communications, Hanoi, Vietnam; g Faculty of Chemistry, Hanoi National University of Education, Hanoi, Vietnam; h Institute for Tropical Technology, Vietnam Academy of Science and Technology, Hanoi, Vietnam; i Vietnam Academy of Science and Technology, Graduate University of Science and Technology, Hanoi, Vietnam

**Keywords:** Polypyrrole, graphene oxide, molybdate doping, nanocomposite, corrosion protection, self-healing protection

## Abstract

In this work, polypyrrole-based nanocomposites doped with graphene oxide, molybdate, and salicylate (PPy/GO/Mo/Sal) were synthesized via *in* *situ* electrochemical polymerization to enhance the anti-corrosion protection performance of polymer coatings. The morphology and structures of the coatings were characterized by SEM, EDX, FTIR, Raman spectroscopy, and XRD. The protection abilities of coatings against corrosion were investigated in 0.1 M NaCl solution with EIS potentiodynamic polarization, salt spray test, and open-circuit potential (OCP) measurements. The results showed that with the presence of both molybdate/salicylate and GO in the PPy matrix, the nanocomposite coating exhibited an excellent protection ability against corrosion for low-carbon steel, better than that with only GO as filler. Compared to the nanocomposites doped with only salicylate or salicylate/GO, the one doped with both molybdate/salicylate and GO exhibited the longest protection plateau (ca. 100 h) on the OCP-time curves with some fluctuation points known as the self-healing action of molybdate dopant. It also resulted in a decrease in the corrosion current (Tafel plots), a higher impedance (Bode plot), and a better protection performance in salt spray tests. In this case, the anti-corrosion ability of the coatings was provided through a barrier and self-healing mechanism.

## 1. Introduction

Nanocomposites are a new generation of materials with high potential for applications in many fields, including at least one nanoscaled component dispersed in a polymer matrix such as epoxy, polyethylene, polypropylene, and conducting polymers [1–3]. Carbon nanotubes [4], graphene oxide [5], titanium oxide [6], and other metal-based nanoparticles [7] are commonly used as additives or fillers in these matrices to form nanocomposites and improve some particular physicochemical properties of the materials for a given application, *e.g.,* corrosion resistance.

Corrosion protection for metals has been intensively studied because metal corrosion leads gradually to metal loss and damage to metallic structures. Although metals are widely used due to their various good properties, they cannot protect themselves in extremely corrosive media, especially active metals such as Fe, Zn, Mg, and so on. One of the most used methods to protect metallic structures is using organic coatings to isolate them from the environment, or more precisely, to cover the metal surface with a barrier film to prevent corrosive species from going through [8,9]. Some coatings can slow down and then stop the development of defects, micropores, or cracks on the coating, so-called ‘self-healing coatings’. Among the organic coatings, conducting polymers (CP) such as polyaniline, polypyrrole, or polythiophene have been used as potential alternatives for the development of new protective coatings with no or less toxicity, good stability, and excellent adhesion to the metal surface. Among CPs, polypyrrole (PPy) has received the most attention from scientists. PPy can be used in many application fields such as sensors, antistatic fabric, aircraft, medical, and electrochromic fields [10–12]. Recently, PPy has been studied for its anti-corrosive properties on metal substrates via different mechanisms including anodic passivation, cathodic protection, controlled inhibitor release, and barrier effect [13–15]. In some cases, with suitable dopants, polypyrrole coating can provide anti-corrosive action via a self-healing mechanism. These dopants can prolong the protection time of coatings for active metals [16–18]. The main problem of PPy is that it cannot be electrochemically formed on the active metal substrate without pretreatment [19–23]. Moreover, due to its low solubility in organic solvents, it is not possible to coat PPy on active metal substrates by brushing or painting. To deal with this difficulty, the most common way is to use PPy nanoparticles as fillers/additives or pigments in the matrix polymers of binders or resins [24–27]. Besides, to improve the barrier effect of coatings, inorganic fillers/additives, especially nanoscaled materials, are often used in combination with organic coatings. Due to their strong interaction with polymer matrices, they can offer a durable corrosion protection action of nanocomposite coatings. In the presence of suitable nanofillers/additives, the coating becomes more compact with lower porosity, which results in an enhancement of the anti-corrosion ability of the coating. Among these inorganic fillers, graphene oxide (GO), which is a graphene sheet with oxygen-containing groups such as epoxide, phenolic, or hydroxyl groups located on its basal plane, emerges as a potential candidate due to its outstanding mechanical, optical, thermal, and electrical properties as well as its high chemical inertness, good thermal stability, and impermeability [28–30]. As a result, GO can be an effective anti-corrosion additive for self-healing coating [31–33].

The combination of polypyrrole and graphene oxide in anti-corrosion coatings has been used in several studies. Amirazodi *et al*. reported that the dispersion of PPy and GO in epoxy resin resulted in a better anti-corrosion performance of nanocomposite coatings on mild steel, which was interpreted by a good dispersion and interaction between PPy, GO, and the epoxy matrix [34]. In another work, a nanocomposite pigment based on zinc phosphate and PPy functionalized graphene oxide (ZGP) was fabricated via *in situ* polymerization and then filled in waterborne epoxy coating for enhancing the anti-corrosion performance of the latter [35]. It was reported that the corrosion protection was significantly improved when embedding a small amount of ZGP, compared to the neat epoxy coating, which was explained by the synergistic protection effect of impermeable GO-PPy nano-sheets and the passive function of zinc phosphate. PPy/GO nanocomposites could also be electrodeposited on 304 stainless steel bipolar plates to form effective coatings against corrosion via an enhanced physical barrier and anodic protection mechanism compared to pure PPy coatings [36].

However, it is known that coatings are difficult to electrodeposit *in situ* on steel due to the dissolution of steel right after applying the current, and therefore steel passivation is expected to be a good approach for this issue. In our previous work, the PPy-based coatings doped with molybdate/salicylate on low-carbon steel were successfully synthesized and characterized. The results revealed that molybdate and salicylate are potential candidates to form passive layers on steel, which inhibit the dissolution of steel during the pyrrole polymerization [37]. Molybdate dopants also play an important role in the enhancement of corrosion resistance of the nanocomposite coatings. It is therefore interesting to investigate to what extent the corrosion protection of steel can be improved by introducing both graphene oxide as filler and molybdate and salicylate as dopants in the polypyrrole matrix. To our knowledge, there are no studies involving the combined use of such fillers and dopants.

The present study aimed to *(i)* synthesize and characterize different nanocomposite coatings on the low carbon steel substrate via an *in situ* electropolymerization process (using Py, GO, salicylate, and molybdate as precursors); *(ii)* measure the electrochemical properties of nanocomposite coatings to investigate their protective effects against corrosion; as well as *(iii)* clarify the role of GO filler and molybdate dopant in the corrosion protection performance of coatings.

## 2. Experimental section

### 2.1. Chemicals

Chemicals used in this study included pyrrole (Py) monomer (98%), sodium salicylate (99.5%), sodium molybdate (98%), and sodium chloride (99.5%), all purchased from Sigma-Aldrich. Pyrrole monomer was purified by distillation prior to use and stored at a low temperature, protected from light. Graphene oxide (GO) was obtained from the Vietnam Academy of Science and Technology.

Commercial low-carbon steel was purchased from TISCO Company, Thai Nguyen, Vietnam. The composition (wt%) was 0.10% C, 0.5 Mn, 0.04 P, and 0.05 S.

### 2.2. Preparation of low-carbon steel substrates

To be used as a substrate, commercial low carbon steel plates (Vietnam) were cut into small pieces of 4 cm × 2 cm × 0.5 cm dimensions, mechanically polished using a series of emery papers (600, 1000, 2000 grit), rinsed with distilled water and ethanol, finally dried with an N_2_ gas stream.

### 2.3. Electrosynthesis of nanocomposites

GO and pyrrole monomers were first well dispersed in distilled water using an ultrasonic bath for 30 min to obtain a homogeneous dispersion. Molybdate and salicylate dopants were then added to this solution with the electrolyte solution composition for three samples M1, M2, and M3 as shown in Table 1. The solution was stirred for 60 min prior to electropolymerization and kept stirring during electropolymerization. A constant current technique (at a current density of 1 mA/cm^2^) was used to form a PPy-based nanocomposite layer on the steel substrate as a working electrode in a three-electrode cell, while the reference electrode was Ag/AgCl and the counter electrode was a Pt sheet. Finally, the nanocomposite samples coated on steel substrates were carefully washed with distilled water and dried in an N_2_ stream.

**Table 1.** Electrolyte solution composition for nanocomposite electrodepositions.

No.	Sample	Pyrrole (mol/L)	Salicylate (mol/L)	GO (mg/L)	Molybdate (mol/L)
1	M1	0.10	0.10	0.00	0.00
2	M2	0.10	0.10	200.00	0.00
3	M3	0.10	0.10	200.00	0.01

### 2.4. Characterization

The synthesized nanocomposite coatings were characterized by physicochemical methods. The morphology and element composition of the composite samples were analyzed using a Scanning Electron Microscope (SEM) with Energy-Dispersive X-ray (EDX) instrument (Hitachi S-4800 FESEM). Fourier Transform Infra-Red (FTIR) spectra were recorded on an IR Prestige-21 Shimadzu in the frequency range of 500–4000 cm^−1^. X-ray diffraction (XRD) was studied in the 2θ range of 20°−80° using a Shimadzu 6100× X-ray diffractometer. The Raman scattering of these materials was performed with a Raman spectrometer HORIBA XploRA Plus at 532-nm excitation.

### 2.5. Corrosion test

Anticorrosive properties of PPy-based nanocomposites-coated low-carbon steel samples were investigated by Open Circuit Potential (OCP), Electrochemical Impedance Spectroscopy (EIS), and Potentiodynamic Polarization. Measurements were carried out in 0.1 M NaCl solution as corrosive media at room temperature. EIS analysis was performed in a frequency range from 100 kHz to 10 mHz with an amplitude of 10 mV using the Zahner workstation (Germany). To obtain Tafel polarization and potentiodynamic polarization curves, the electrode potential was scanned from −400 mV to 600 mV at a scan rate of 1 mV/s also using the Zahner workstation.

A salt spray test was also conducted using Q-FOG CCT 600 salt spray test instrument to test the stability of the PPy-based nanocomposites in 50 mg/L NaCl at a temperature of 35°C and a pressure of 100 kPa.

All electrochemical measurements were performed using a three-electrode cell in which steel substrate samples with an exposed area of 1 cm^2^ were used as the working electrode, Pt sheet as the counter electrode, and Ag/AgCl as the reference electrode.

## 3. Results and discussion

### 3.1. Electropolymerisation of nanocomposites

**Figure 1.**
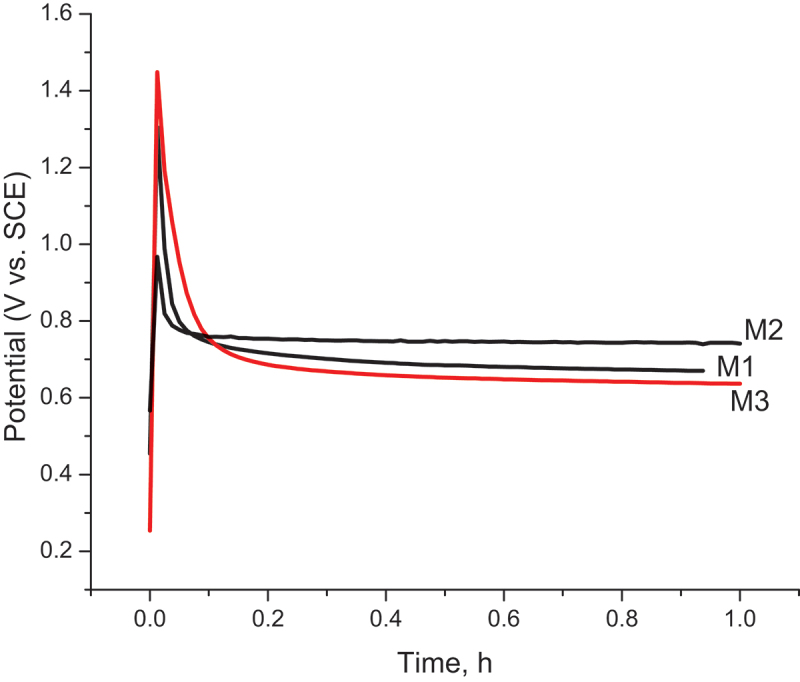
Potential-time curves of Py electropolymerization at 1 mA/cm^2^ in M1, M2, and M3 samples.

Potential-time curves (Figure 1) were obtained during the electropolymerization of PPy nanocomposites at a current density of 1 mA/cm^2^.

It can be seen from Figure 1 that, for all samples, the potential increased sharply right after applying the current and then dropped down and stabilized at the potential of pyrrole oxidation. No induction period has been observed for any of the experiments, which means that GO appeared to have no effect on the PPy deposition on low-carbon steel. This behavior is often observed for the electrodeposition of PPy on inert or passive electrodes. The oxidation potential of pyrrole was slightly different between the three samples. In the presence of molybdate in the electrolyte solution, the oxidation of pyrrole in the M3 sample was the lowest compared to the one in M1 and M2 samples.

Recently, salicylate and molybdate dopants have been considered good inhibitors. They can passivate the low-carbon steel before electropolymerization occurs. As depicted in Figure 1, molybdate showed a better inhibitory performance. The passive layer of molybdate seemed to be thicker, so the current needed for nucleation was higher. In the electric field, GO moved toward the anode electrode due to the charge repulsion of functional groups containing oxygen in GO sheets and then incorporated into the PPy structure [38]. This resulted in a lower oxidation potential of pyrrole in the M2 sample than in the M1 sample. In other words, PPy-based nanocomposites were successfully formed on steel substrates with GO, molybdate, and salicylate.

### 3.2. Morphological and structural studies

#### 3.2.1. Morphology and element composition

3.2.1.

**Figure 2.**
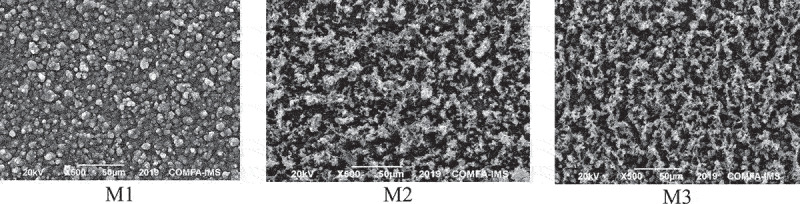
SEM pictures of M1 (a, b), M2 (c, d), and M3 (e, f) samples with different magnification (please add more 3 pictures as in the word file in the last mail).

The morphology of the three coated samples M1, M2, and M3 was investigated with SEM, and the results are shown in Figure 2. All SEM images exhibit a uniform coverage of coatings on the substrate surfaces with different morphologies, indicating a good adhesion of all coatings on the substrate via the in-situ electrodeposition process with no sign of film cracking. While the M1 sample showed a typical compact cauliflower-shaped structure of the PPy morphology [39–41], the nanocomposite samples M2 and M3 showed completely different morphologies.

The difference in the morphology of M2 and M3 samples to M1 sample may be due to the dispersion in the PPy matrix. The structure of M2 and M3 nanocomposites seem more porous thanks to the dispersion of GO sheets. At higher magnification (Fig. 2d, and Fig. 2f), the cauliflower structure of PPy was absence. The presence of GO sheets in the polymerization process could cause the discontinuity in the PPy matrix. In adđition, the doping mobybdate anions to the nanocomposite coating cause a negligible change in the morphology of M3 as compared to that of M2. However, it still sees the morphology of M3 become more compact than that of M2. It could be explained that the molybdate anions could enhance the passivation of the stelll substrate so that homogeneity of the PPy film was improved. It can be concluded that GO has been well dispersed in the polymer matrix and the molybdate doping has improved the appearance of nanocomposites.

**Figure 3.**
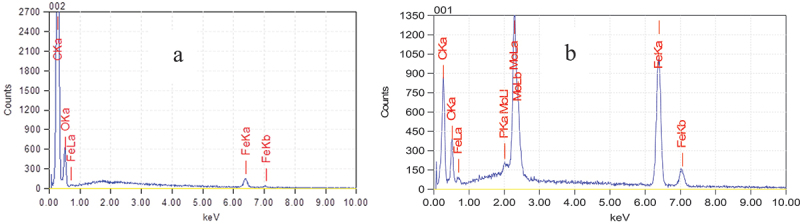
EDX spectrum of M1 (a) and M3 (b).

EDX results (Figure 3b) allowed confirming the above SEM results that molybdenum (Mo) was doped into PPy nano-coating, about 21% by mass, while it could not be detected in the sample M1 (Figure 3a).

#### 3.2.2. X-ray diffraction

**Figure 4.**
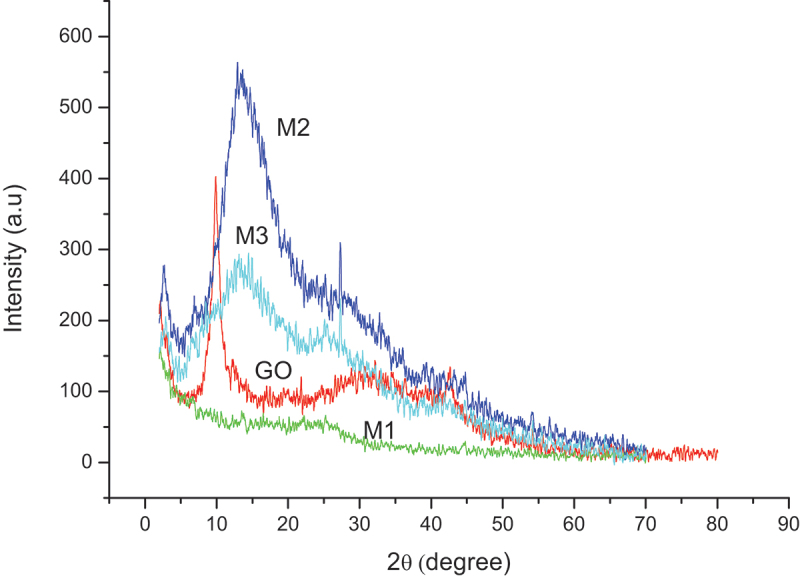
XRD spectra of GO, M1, M2, and M3 samples.

XRD patterns of GO, PPy, and nanocomposites are shown in Figure 4. The results reveal an amorphous structure of polypyrrole (M1) displayed by a broad band at around 2θ = 25° [40] and the crystal structure of graphene oxide (GO) with a strong diffraction peak at 2θ = 9.98° corresponding to the reflection plane of (002). This 2θ angle reflects the interlayer spacing in GO, which is related to the hydration degree of GO [42], calculated via Bragg’s equation (nλ = 2d sinθ) to obtain d = 0.88 nm.

For the M2 and M3 samples, the intercalation of PPy into GO nanosheets resulted in a broadening and a shift of the diffraction peak to larger angles, from 2θ = 9.98° to about 13°, which indicated a decreasing interlayer space of GO nanosheets, about 0.7 nm. In addition, with molybdate doping (M3), a decrease in the intensity of the diffraction peak of GO implies a better wrapping of GO by PPy spheres. However, the intercalation of PPy into GO interlayers was not thorough since the diffraction peaks of GO still appear. A similar result has been observed by Zhu et al. with the matrix containing GO and PPy at mass ratios of 1:1 and 2:1 [43].

### 3.3. Raman Spectra

**Figure 5.**
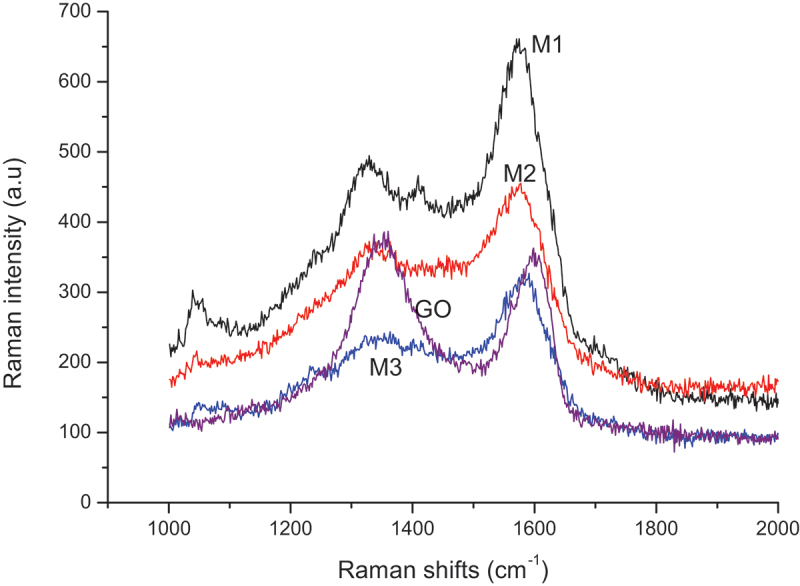
Raman spectra of GO, M1, M2, and M3.

Scattering Raman spectra of neat GO, M1, M2, and M3 are shown in Figure 5.

In the range of Raman shifts from 1000 to 2000 cm^−1^, all samples exhibit a D-band at about 1340 cm^−1^, and a G-band at about 1580 cm^−1^ which are typical for most carbon nanostructures [44]. The formation of the D band is related to the defects and edges of GO and is caused by C-N bonds of PPy [35]. Moreover, the intensity ratio of these two bands (I_D_/I_G_) can be used as an indirect tool to estimate the disorder of graphene within different coatings (neat GO, M2, and M3). The M3 nanocomposite showed the lowest intensity ratio (I_D_/I_G_ = 0.73) compared to M2 (ID/IG = 0.8) and the neat GO (ID/IG = 1.03). This result can imply an effect of polypyrrole to fill the vacancies in the GO structure, which led to lower defect sites [45,46].

### 3.4. Fourier transform infrared analysis

**Figure 6.**
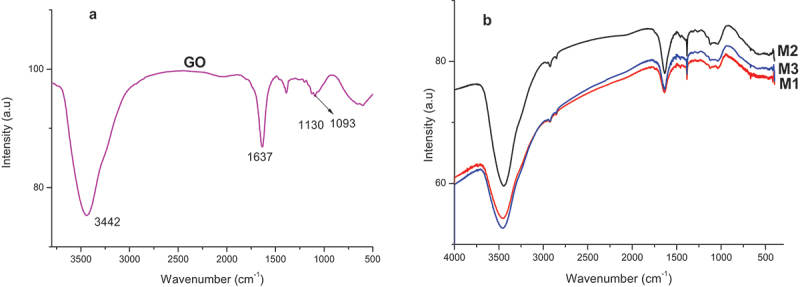
FTIR spectra of GO (a) and M1, M2, and M3 (b).

Chemical structure and functional groups present in the samples were studied by FTIR spectroscopy. FTIR spectra for the different samples are shown in Figure 6, all of which exhibit a broad band at around 3440 cm^−1^ assigned to the O-H stretching of water molecules absorbed in the GO. Besides, Figure 6a displays all characteristic absorption bands of GO structures with oxygen-containing functional groups such as 1637, 1400, and 1093 cm^−1^ corresponding to C=O stretching, C-O stretching vibration, and O-H deformation, respectively [40,41,43]. Meanwhile, Figure 6b displays FTIR spectra of PPy-based nanocomposites with typical peaks of pyrrole structure found at 1474, 1262, and 1123 cm^−1^. Due to pyrrole ring vibration, C–H in-plane vibration, and C–N stretching vibration, respectively. Moreover, a decrease in the intensity of the C=O stretching band in nanocomposites reveals a formation of hydrogen bonds between the carboxyl group of GO and the -NH group of PPy. These results confirm that PPy was formed successfully in the nanocomposite with GO as fillers.

### 3.5. Corrosion protection performance

**Figure 7.**
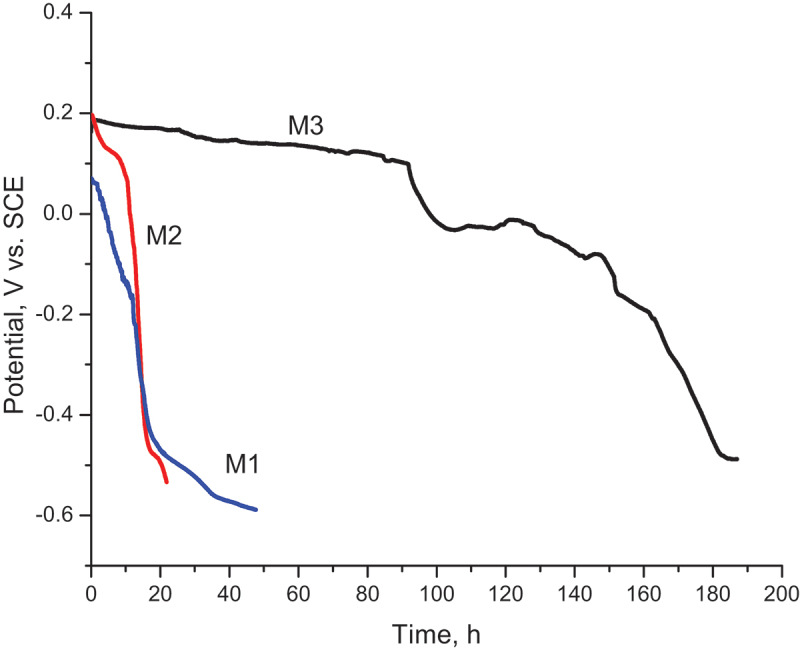
OCP-time curves of M1, M2, and M3 in 0.1 M NaCl solution.

The anti-corrosion ability of nanocoatings was investigated by electrochemical methods. OCP–time curves were obtained in 0.1 M NaCl as presented in Figure 7.

It can be clearly seen from the OCP plots that in the presence of GO, the potential of nanocomposite-coated steel (M2, M3) remained in the positive region much longer than that of pure PPy coating (M1) under the same testing condition. M3 exhibited the longest plateau of potential, and the fluctuation of potential could only be seen till the end of the immersion.

At the beginning of the test, the noble shift in the potential of the nanocomposite coatings M2 and M3 is more noticeable than the pure PPy coating (M1). OCP of M1 was gradually decreased and approached the corrosion potential of bare steel after 15 h of testing. After this time, Ppy coating could not protect the steel substrate anymore. However, nanocomposite coatings M2 and M3 could provide a positive potential for a longer time. The noble shift of OCP and potential plateau are indicators for the redox-induced passivation of the steel surface. Due to the layer structure, GO plays an important role in the corrosion protection of steel by providing a barrier effect between corrosive species and steel surfaces. 

However, the barrier ability of the composite coating reduces obviously after the coating is damaged. When the substrate was corroded, OCP of Ppy coating dropped immediately. Compared to M1 and M2, M3 had the longest protection plateau of potential. Its OCP remained in the positive region for ca. 100 h of dipping in NaCl solution. The fluctuation of OCP could be seen.  

After 100 hours immersion, when a defect was introduced into the PPy-coated steel, the potential decreased and activation of the coated steel started. After that, the potential was recovered to the passive region. This process of activation and passivation continued to the end of the experiment. So that, it resulted in the fluctuation points of OCP. It is known that self-healing action. This action could not be seen for M1 and M2. Molybdate was assigned to this effect.

It can be concluded that GO and molybdate dopants in nanocomposite coatings have provided a synergistic effect on steel corrosion protection, which strongly improves the anticorrosion ability of the nanocomposite coating.

**Figure 8.**
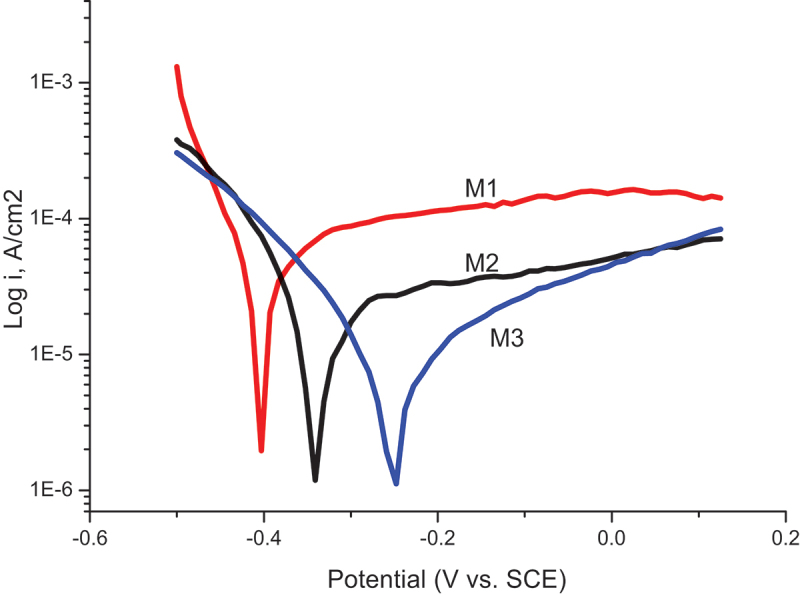
Tafel plots of M1, M2, and M3 in 0.1 M NaCl solution, scan rate 10 mV/s.

The Tafel plots were also recorded when the samples M1, M2, and M3 were immersed in 0.1 M NaCl solution, as shown in Figure 8. It can be clearly seen that the nanocomposite coatings resulted in decreased corrosion currents compared to the pure PPy coating, especially with the doping of molybdate. Similarly, the corrosion potential was also shifted to noble values when both GO and molybdate were incorporated into the polymer matrix. The values of corrosion current (I_corr_) and potential (E_corr_) of the samples M1, M2, and M3 are calculated from the Tafel curves and presented in Table 2.

**Table 2.** Corrosion current and potential of the samples.

Sample	I_corr_ (mA/cm^2^)	E_corr_ (V)
M1	5.8 × 10^−5^	−0.40
M2	2.11 × 10^−5^	−0.33
M3	1.03 × 10^−6^	−0.24

In general, high corrosion potential and low corrosion current are indicative of good anti-corrosion protection. As shown in Table 2, the polarization results reveal an improvement in protection ability against corrosion obtained with the incorporation of GO into the PPy matrix (M2, M3). The best protection performance was achieved with the incorporation of both GO and molybdate (M3).

**Figure 9.**
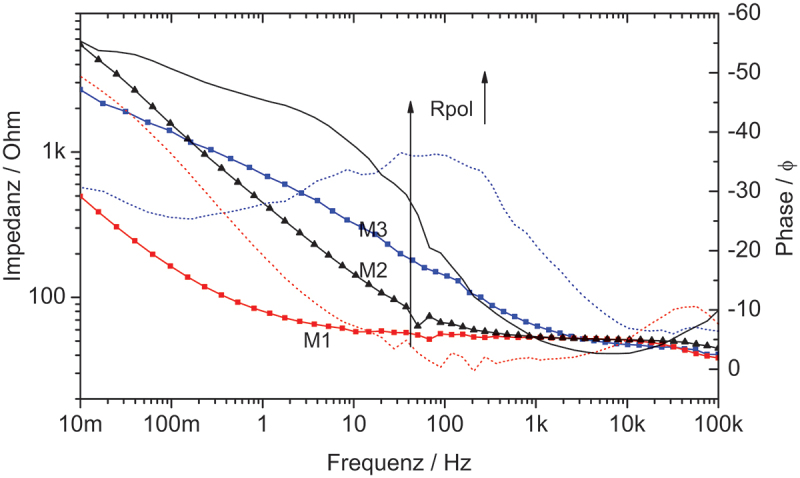
EIS spectra of M1, M2, and M3 in 0.1 M NaCl solution.

It can be observed from the Bode plots in Figure 9 that, in the presence of molybdate in GO/PPy film, the impedances of the M3 film were higher than that of M2 after 30 min of immersion. The polymer film resistance (R_pol_) could be 500 Ω after immersion. The highest impedance obtained for the M3 sample indicates the stability of the coating and good anti-corrosion performance in saline media. Meanwhile, the PPy coating (M1) was conductive and could not be a good barrier for corrosion protection.

**Figure 10.**
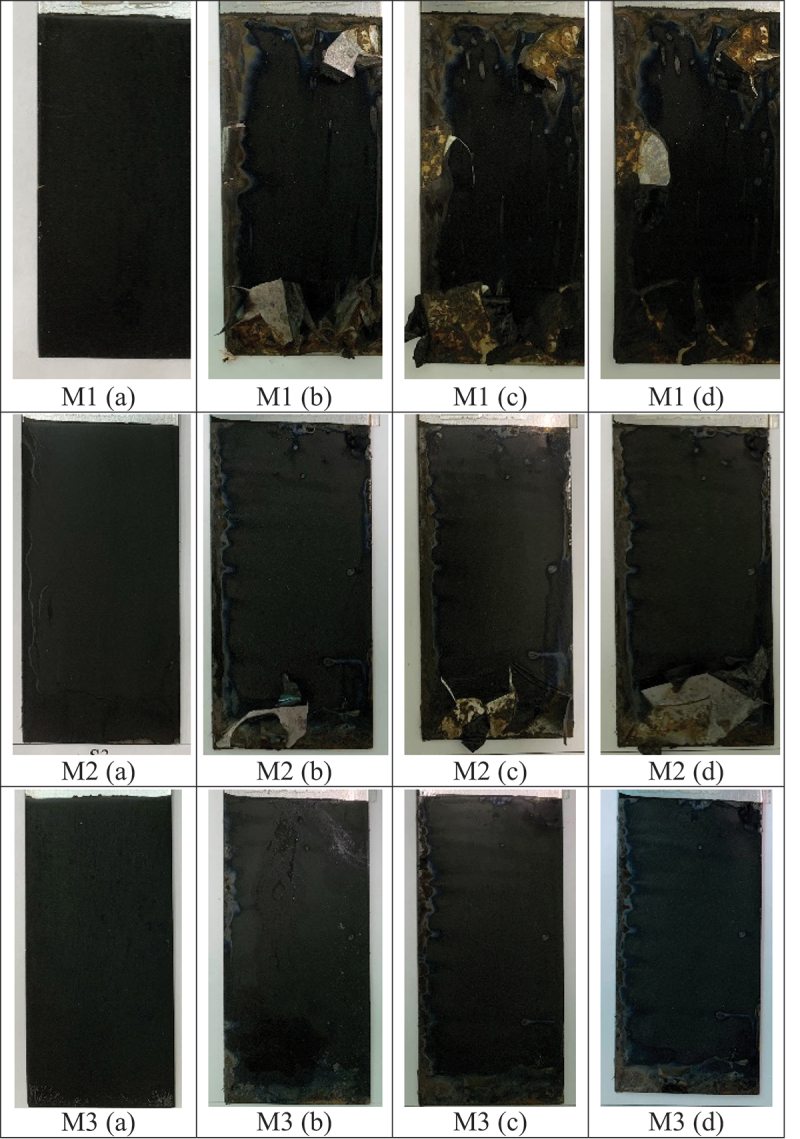
Pictures of samples M1, M2, and M3 in the salt spray test chamber after 0 h (a), 5 h (b), 10 h (c), and 15 h (d).

Pictures of the samples M1, M2, and M3 after spending a certain time in the salt spray test chamber are shown in Figure 10. Some changes in the coatings associated with the corrosion could be observed for all samples after a 5-h exposure in salt media. However, the difference in corrosion degree was significant among them. Delamination of the film could be seen in the M1 and M2 samples where the decomposition of the M1 sample occurred strongly after 5 h of exposure and it was damaged totally after 15 h. In the presence of GO, the protection ability of the film could be better. The damaged area of the M2 film was smaller than that of M1. The M3 nanocomposite film even remained stable after 15 h, staying in the test chamber without any delamination. It is clear that Mo and GO additives in the M3 film provided a better protection ability on the steel substrate.

The results of all the above corrosion tests reached the same conclusion. GO and molybdate as additives/dopants have promoted the anticorrosion performance of nanocomposite coatings on low-carbon steel in salt media. GO layer structure has worked as a good barrier to prevent the penetration of corrosive species through the coating to the steel surface, which generates the passivation of the latter. The π-π stacking of graphene along with the PPy backbone could produce a dense coating that protected the steel substrate from the penetration of electrolytes as the barrier effect. In addition to that, molybdate dopant along with GO incorporation into PPy matrix not only reinforces the barrier layer but also improves the self-healing ability of the steel substrate.

## 4. Conclusion

Polypyrrole-based nanocomposites doped with both salicylate/molybdate and graphene oxide (GO) were successfully synthesized as nano-coatings on low-carbon steel via an *in situ* process of electropolymerization for which the substrate did not require any special pretreatment. Their morphological and structural properties were characterized by SEM, EDX, XRD, FTIR, and Raman spectroscopy. Compared to the salicylate-doped PPy film, the morphology of the nanocomposites changed when GO was incorporated into polymer coatings, and their electrochemical properties were also improved, especially their ability to protect low-carbon steel against corrosion. More impressively, the doping of both salicylate/molybdate and GO resulted in a better wrapping of GO by PPy spheres, meaning a better dispersion of GO in the polymer matrix. Consequently, an excellent anticorrosive ability was achieved with this doping, proven through the results of OCP, EIS, Tafel plots, and salt spray tests in a saline environment. This result can be explained by the synergistic protection of GO and self-healing PPy in the presence of salicylate/molybdate dopants.

## Data Availability

The data used to support the findings of this study are included in the article.
